# Connections Between ETV6-Modulated Genes: Identification of Shared Features

**DOI:** 10.4137/cin.s556

**Published:** 2008-04-21

**Authors:** Gino Boily, Patrick Beaulieu, Jasmine Healy, Daniel Sinnett

**Affiliations:** 1 Division of Hematology-Oncology, Charles-Bruneau Cancer Center, Research Center, CHU Sainte-Justine, Montreal, Quebec, Canada; 2 Department of Pediatrics, Faculty of Medicine, University of Montreal, Montreal, Quebec, Canada

**Keywords:** ETV6, ets member, transcription factor, microarrays, leukemogenesis

## Abstract

Accumulating genetic and functional evidence point to ETV6 as being the tumour suppressor gene targeted by the deletions at chromosome 12p12-13 found in various cancers, particularly childhood leukemia. ETV6 is a ubiquitously expressed transcription factor (TF) of the ETS family with very few known targeted genes. We recently compiled a list of 87 ETV6-modulated genes that can be classified into a number of subgroups based on their coordinated expression patterns. In the present report, we hypothesized that genes presenting a similar profile of modulation could also share biological features, promoter sequence similarities and/or, common transcription factor binding sites (TFBSs). Using an exploratory approach based on hierarchical clustering of expression data, Gene Ontology (GO) terms, sequence similarity and evolutionary conserved putative TFBSs, we found that many genes presenting a similar expression profile also share biological features and/or conserved predicted TFBSs but rarely show detectable promoter sequence similarities. We also calculated the proportion of ETV6-modulated genes that have any conserved TFBSs of the Jaspar database in their regulatory sequence and compared these proportions to those calculated for two other gene lists, ETV6 non-modulated and ETS-regulated. We found that the NF-kB, c-REL and p65 TFBSs, which all bind TFs of the REL class, were under-represented among the ETV6-modulated genes compared to the ETV6-non-modulated genes, while the Broad-complex 1 TFBS appeared to be over-represented. NF-Y and Chop/cEBP TFBSs were over-represented in the promoters of ETV6-modulated genes compared to ETS-regulated genes. These analyses will help direct further studies intending to understand the role of ETV6 as a transcriptional regulator and aid in constructing the ETV6-regulatory gene network.

## Background

Transcription is one of the main levels of regulation of gene expression. Amongst the players involved in this process, transcription factors (TFs) are trans-elements regulating the rate of transcription of particular genes by binding to cis-acting elements known as transcription factor binding sites (TFBSs). However, the whole repertoire of genes targeted by a single TF is not necessarily modulated at the same time or in the same direction (Alvarez et al. 2003).

The mechanisms underlying the co-regulation of subsets of genes are still largely elusive. It is currently thought that particular TFs act together in different combinations and that, depending on these combinations, they co-ordinately regulate particular groups of genes.(e.g. Nishio et al. 1993; Christoffels et al. 1998). It is also suggested that such combinations of TFs can be found as modules in the promoter of co-regulated genes (Klingenhoff et al. 2002). Based on our actual understanding of transcription, it can be hypothesized that some TFs act together in the regulatory region of a limited group of genes leading to their co-regulation and eventually, affecting biological processes at the levels of the cell, tissue and organism as a whole. If this holds true, one could expect that co-regulated genes will share common features in their promoter.

In earlier studies, we and others provided evidence that ETV6, a TF of the ETS family, was inactivated in childhood pre-B acute lymphoblastic leukemias (Kim et al. 1996; Cave et al. 1997; Montpetit et al. 2002; Montpetit et al. 2004). In an attempt to further understand the role of this TF in leukemogenesis, we recently identified 87 putative ETV6-modulated genes using an inducible cell system and microarray analysis in a time course study (Boily et al. 2007). Although it is not yet known if these genes are all directly targeted by ETV6, their expression intensity was shown to vary upon the induction of ETV6. These genes can be grouped in a number of clusters based on the similarity of their expression profiles. The nature of the underlying mechanisms responsible for the modulation of specific genes in the same order of magnitude or pattern in time is an interesting question that still remains to be answered. Though it is still not known if genes with similar expression profiles are necessarily associated to similar biological features, we believe that the combinatorial use of microarray data and Gene Ontology (GO) descriptive biological terms (Ashburner et al. 2000) will provide insights into the particular functions of gene products as suggested by previous studies (e.g. Abba et al. 2005; Patil et al. 2005).

In this study, we used the ETV6-modulated gene list along with computer-based tools such as ConSite, Dialign as well as various clustering methods to address the following questions: first, do genes with similar modulation profiles share 1) biological features, 2) sequence similarity in their promoter, and/or 3) related TFBSs?; and secondly, are certain TFBSs over- or under-represented in ETV6-modulated genes compared to ETV6-non-modulated and ETS-regulated genes? The methodological approaches presented in this exploratory study should be useful in providing the groundwork for future studies aiming at deciphering the functional and regulatory networks of TFs.

## Results

**Figure 1:**
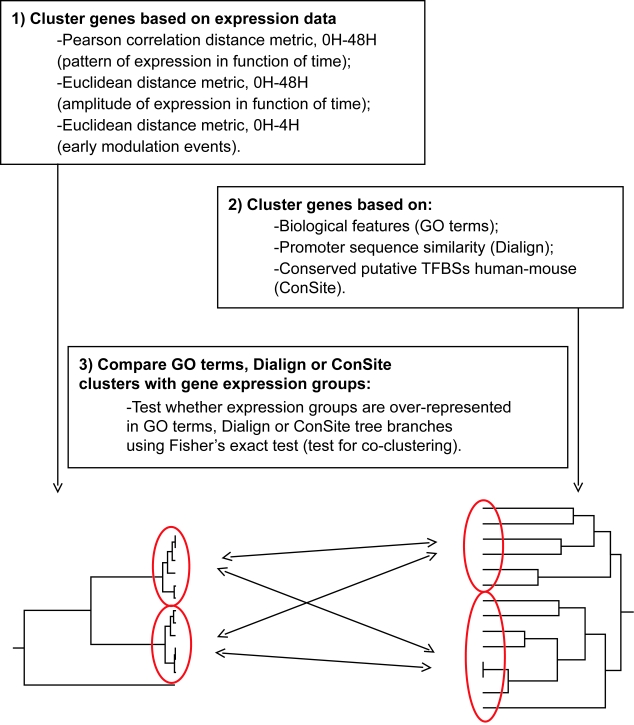
Schematic representation of the three-step clustering strategy **Step 1:** ETV6-modulated genes were clustered according to their expression profiles in time. **Step 2:** The same genes were clustered based on their biological features using GO terms, their promoter sequence similarities using Dialign or the predicted human-mouse conserved TFBSs found in their corresponding promoters using ConSite. **Step 3:** Expression groups obtained in Step 1 were statistically tested for over-representation in any branches of either tree generated in Step 2.

Taking advantage of an ETV6 inducible system and of microarray analyses, we recently reported a list of 87 genes modulated in time (48 h) after the induction of ETV6 (Boily et al. 2007). Accordingly, these genes can be classified into a number of groups based on their modulation profiles. To better understand the underlying mechanisms responsible for the observed co-modulation of these genes, we performed association studies using a three-step gene clustering strategy (Fig. 1) based on the assumption that genes with similar modulation profiles might share biological features (GO terms), conserved TFBSs and/or promoter sequence similarities. In other words, we hypothesize that genes with similar expression modulation profiles will share common biological and/or structural features and therefore will cluster together in our analyses.

**Figure 2:**
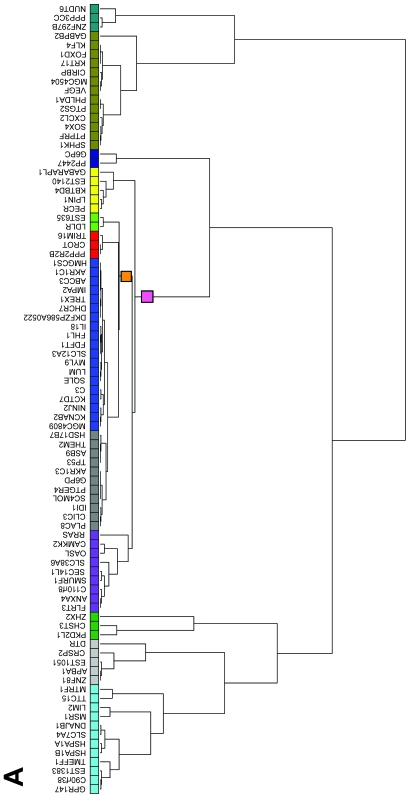

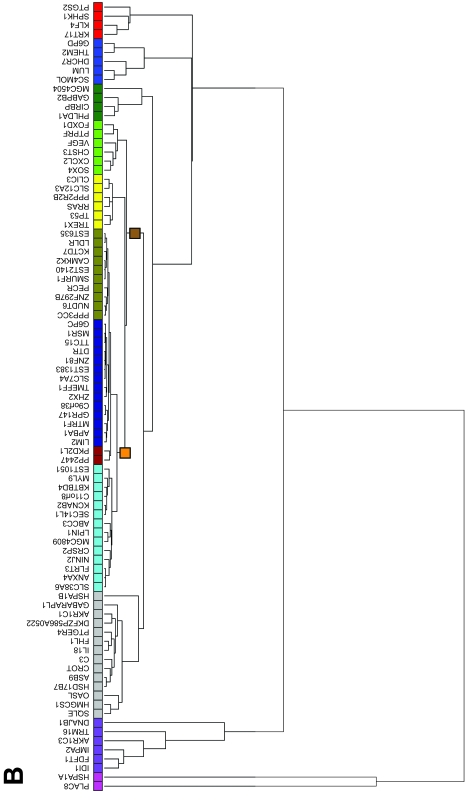

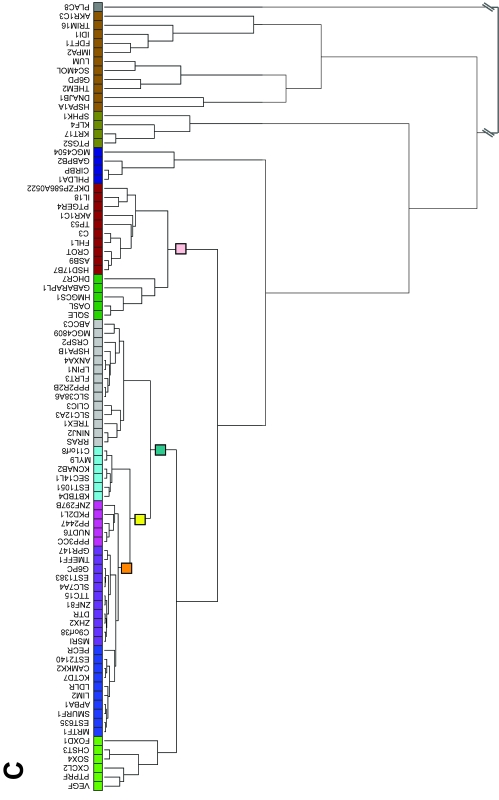
Hierarchical clustering of ETV6-modulated genes by expression profiles ETV6-modulated genes were clustered based on their expression profiles at 0 h, 4 h, 12 h and 48 h (**A** and **B**) or 0 h and 4 h (**C**) after induction of ETV6 (Boily et al. 2007). Clusterings were performed using either Pearson correlation distances (**A**) or Euclidean distances (**B** and **C**). The Pearson correlation metric brings together genes with similar changes in the pattern (direction) of expression vs time whereas the Euclidean distance metric brings together genes with similar changes in intensity of expression vs time. The trees were divided into groups of genes based on the distance between branches and a colour was assigned to every gene according to its respective group (colour below gene symbols). When individual groups were relatively close together, another colour was assigned to the node joining their branch (coloured squares on the tree branches themselves) and these groups were considered both individually and in combination in the analyses.

**Figure 3:**
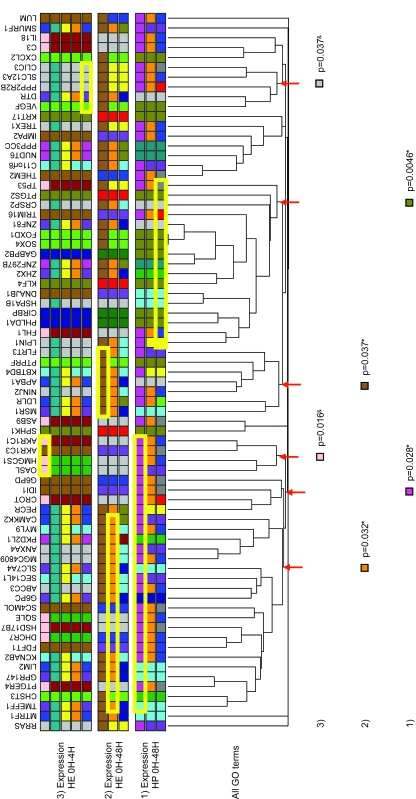
Hierarchical clustering of ETV6-modulated genes by GO terms and associations with expression groups A hierarchical tree of ETV6-modulated genes was constructed using all the GO terms associated to the genes analysed. Coloured squares below gene symbols identify the expression group of the corresponding gene as determined by hierarchical clustering in Figure 2: 1) Pearson correlation distances at 0 h, 4 h, 12 h and 48 h (HP 0 h–48 h) (Fig. 2A); 2) Euclidean distances at 0 h, 4 h, 12 h and 48 h (HE 0 h–48 h) (Fig. 2B) and; 3) Euclidean distances at 0 h and 4 h (HE 0 h–4 h) (Fig. 2C). Expression groups are shown in several rows to represent all the possible group sub-divisions: the bottom row of squares shows the colours that assign each gene to its smallest expression group (colours just below the gene symbols in Fig. 2) and the rows above show the colours assigned to each gene after some close expression groups have been merged (e.g. grey, blue, red and green in Figure 2A are merged in a larger orange branch). Values at the bottom are Fisher’s exact p-values assigned to the node marked by the arrow on the branch above and the expression clustering methods are indicated on the left (numbers). The colour assigned to the p-value is indicative of the group that is over-represented in the branch. The yellow rectangles around the coloured squares delineate the genes that belong to a node where a significant over-representation is observed. FDR correction for multiple testing: *: significant when FDR = 5%; ^+^: significant when FDR = 10%; &: significant when FDR = 15%.

In order to group the genes according to their modulation of expression over time, we chose hierarchical clustering (HC) since, as opposed to other methods like K-means clustering, the resulting trees do not change over repeated analyses with the same data. The expression trees were then divided into expression groups and a colour code was assigned to each group (Fig. 2). Two different distance metrics and two datasets were used to build three trees, each of them reflecting a conceptually different type of co-modulation (see Fig. 2 and Methods). Note that depending on the distance metric used, the shape of the tree and the genes found within each group may be different, as one would expect (Fig. 2). To determine whether any of these expression groups shared specific biological features we used Gene Ontology (GO) terms. The GO project is a collaborative effort to describe gene products in terms of three organizing principles: 1) biological processes, 2) cellular components, and 3) molecular functions (Ashburner et al. 2000). Binary matrices were built with the ETV6-modulated genes and GO terms from 1, 2 or all 3 of the organizing principles (see Methods) and these matrices were used for hierarchical clustering, resulting in trees in which genes sharing GO terms were closer together (Fig. 3). To determine whether genes with similar expression profiles also shared common biological features, GO term trees were compared to the expression groups (see Fig. 2) by assigning an expression group colour code to every gene in the GO term trees (Fig. 3). Using the Fisher’s exact test, we statistically determined whether expression groups were over-represented in any node of the GO term trees (significant associations are depicted at the bottom of Figure 3 and Supplementary Table 1S). Multiple testing corrections were performed using the False Discovery Rate (FDR) since the flexibility of this method is well suited for the exploratory nature of our study (Supplementary Table 1S). We found that several branches of the GO tree were associated with particular expression groups (Fig. 3), suggesting that genes sharing biological features are often co-modulated under similar profiles.

In addition, we also built trees with terms corresponding to each of the GO organizing principles (molecular function, biological process and cell component) taken independently or in a pair-wise combination. A summary of the resulting significant associations are listed in Supplementary Table 1S. Interestingly, clustering based on the molecular function GO organizing principle gave rise to fewer associations than did the other two organizing principles (Supplementary Table 1S). To visualize which genes most frequently co-clustered together, we built a binary matrix with genes as one component and association groups by GO terms as the other component (every group in Supplementary Table 1S was attributed a group number). This matrix was then used to build a hierarchical tree where genes most often co-clustering together by both similar expression pattern and biological features were found closer together in the tree (Supplementary Figure 1SA). The resulting tree clearly showed evidence of a number of genes that appeared to always co-clustered together: MGC4809, ANXA4 and MYL9; IL18 and C3; VEGF and CXCL2; PPP3CC and NUDT6; ZNF297B and TP53; ZNF81 and ZHX2; NINJ2 and FLRT3; SOX4 and FOXD1 and PHLDA1 and CIRBP. On the other hand, some genes were never part of any association, such as C9orf38, FHL1 and IMPA2 for example (Supplementary Figure 1SA). Overall, this tree suggests that genes close together in the hierarchy may be co-modulated by ETV6 and other overlapping regulatory components and participate in related biological features/functions.

To determine the nature of the GO terms responsible for the co-clustering of genes by expression profile, we selected the terms that were related to at least two genes in every association groups (Supplementary Table 1S). Supplementary Figure 1SB presents the terms involved in the associations by frequency of occurrence. Though some terms had a more general connotation such as “nucleus”, “integral to membrane” or “microsome”, others were more explicit, such as “cholesterol biosynthesis”, “calcium ion binding” or “immune response”. In an independent analysis we selected the GO terms of the genes that were never part of any association despite the fact that they were shared by a certain number of genes. The following GO terms were those that were shared by at least two genes in the analysis: hydrolase activity (8 genes), signal transduction (6 genes), magnesium ion binding (5 genes), membrane fraction (4 genes), neurogenesis (3 genes), cell differentiation (3 genes), muscle development (3 genes), molecular function unknown (2 genes), biological process unknown (2 genes), extracellular matrix (2 genes), mitochondrion (2 genes) and intracellular signalling cascade (2 genes). These analyses suggests that the GO terms associated with the clustering of genes refer to biological concepts that were linked to the observed co-modulation of expression whereas terms that were not involved in gene clustering referred mostly to biological concepts that are less likely to be linked to co-modulation.

**Figure 4:**
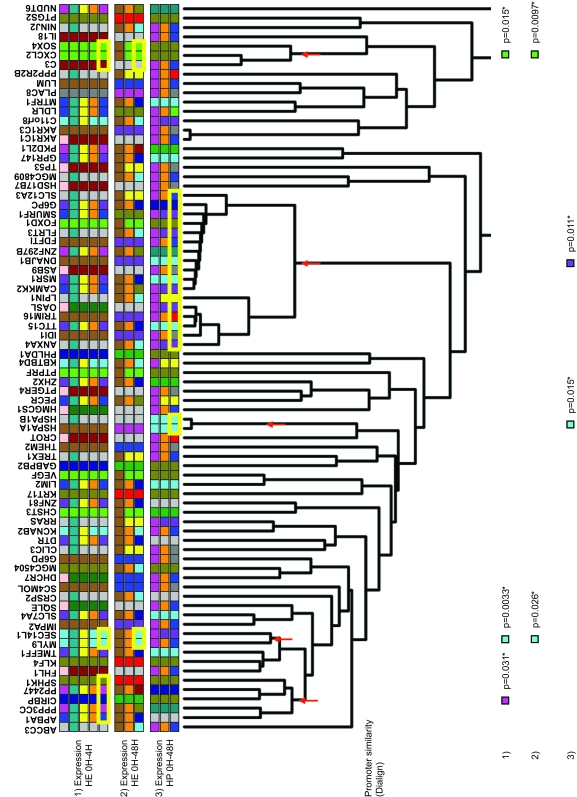
Hierarchical clustering of ETV6-modulated genes by promoter sequence similarity and associations with expression groups A hierarchical tree of ETV6-modulated genes was constructed based on their promoter sequence similarities using Dialign and Phylip programs. Coloured squares below gene symbols identify the expression group of the corresponding gene as determined by hierarchical clustering in Figure 2: 1) Pearson correlation distances at 0 h, 4 h, 12 h and 48 h (HP 0 h–48 h) (Fig. 2A); 2) Euclidean distances at 0 h, 4 h, 12 h and 48 h (HE 0 h–48 h) (Fig. 2B) and; 3) Euclidean distances at 0 h and 4 h (HE 0 h–4 h) (Fig. 2C). Expression groups are shown in several rows to represent all the possible group sub-divisions: the bottom row of squares shows the colours that assign each gene to its smallest expression group (colours just below the gene symbols in Fig. 2) and the rows above show the colours assigned to each gene after some close expression groups have been merged (e.g. grey, blue, red and green in Fig. 2A are merged in a larger orange branch). Values at the bottom are Fisher’s exact p-values assigned to the node marked by the arrow on the branch above and the expression clustering methods are indicated on the left (numbers). The colour assigned to the p-value is indicative of the group that is over-represented in the branch. The yellow rectangles around the coloured squares delineate the genes that belong to a node where a significant over-representation is observed. FDR correction for multiple testing: *: significant when FDR = 5%.

To assess whether genes with similar expression profiles also shared promoter sequences we clustered ETV6-modulated genes according to their promoter sequence (1 kb upstream of exon 1) similarities using the Dialign alignment tool (Morgenstern et al. 1998), and then compared this Dialign tree with the expression groups obtained in Figure 2. Considering the same rationale as above, if co-modulated genes share sequence similarities in their promoters, we would expect certain expression groups (represented by the colour codes) to be over-represented in Dialign branches. One large branch had an over-representation of genes from the HP 0 h–48 h expression clustering (purple group, Fig. 4). Other associations implicating smaller branches and a smaller number of genes were also found (Fig. 4). We reasoned that this low degree of association might be due to the, sometimes, imperfect identification of transcriptional start sites (TSS) as reported in public databases. An oligo-capping method has recently been developed to obtain full length cDNAs and thousands of cDNA sequences have been deposited in the Database of Trancriptional Start Sites (DBTSS) (Ota et al. 2004). Using 1.5 kb upstream of these alternative TSSs, we constructed another Dialign tree. Some new associations were found but still did not involve many branches of the tree (data not shown). Overall, these analyses suggest that a very limited number of co-modulated genes share extended promoter sequence similarities, at least as detected by our approach.

**Figure 5:**
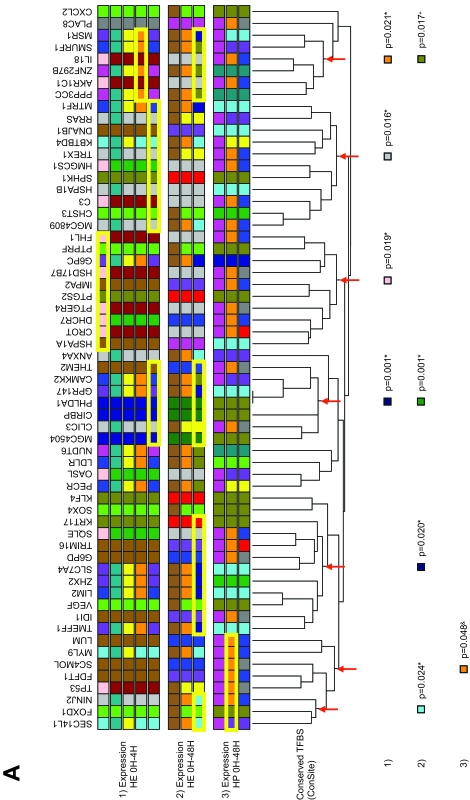

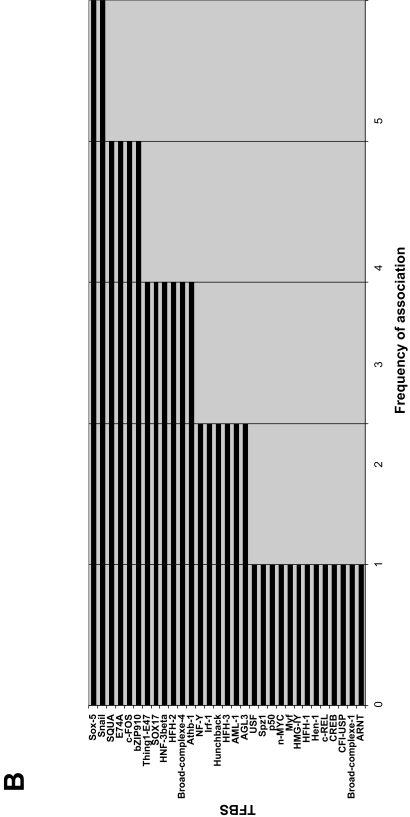
Hierarchical clustering of ETV6-modulated genes by conserved putative TFBSs found in their promoter and associations with expression groups **A.** A hierarchical tree of ETV6-modulated genes was constructed based on conserved putative TFBSs found in their promoter using ConSite. Coloured squares below gene symbols identify the expression group of the corresponding gene as determined by hierarchical clustering in Figure 2: 1) Pearson correlation distances at 0 h, 4 h, 12 h and 48 h (HP 0 h–48 h) (Fig. 2A); 2) Euclidean distances at 0 h, 4 h, 12 h and 48 h (HE 0 h–48 h) (Fig. 2B) and; 3) Euclidean distances at 0h and 4h (HE 0 h–4 h) (Fig. 2C). Expression groups are shown in several rows to represent all the possible group sub-divisions: the bottom row of squares shows the colours that assign each gene to its smallest expression group (colours just below the gene symbols in Fig. 2) and the rows above show the colours assigned to each gene after some close expression groups have been merged (e.g. grey, blue, red and green in Fig. 2A are merged in a larger orange branch). Values at the bottom are Fisher’s exact p-values assigned to the node marked by the arrow on the branch above and the expression clustering methods are indicated on the left (numbers). The colour assigned to the p-value is indicative of the group that is over-represented in the branch. The yellow rectangles around the coloured squares delineate the genes that belong to a node where a significant over-representation is observed. **B**. Terms involved in the associations were counted and plotted by frequency of occurrence FDR correction for multiple testing: *: significant when FDR = 5%; ^+^: significant when FDR = 10%; ^&^: significant when FDR = 15%.

Next we wanted to test the possibility that ETV6-modulated genes share common TFBSs. Since the short and degenerate nature of TFBSs leads to the prediction of a high rate of false positive putative binding sites (Wasserman and Sandelin, 2004), we applied a phylogenetic footprinting approach that is based on the premise that functionally important elements should be conserved between species or otherwise lost with evolution (reviewed in Zhang and Gerstein, 2003). To this effect, we used the ConSite software (Sandelin et al. 2004) to identify putative TFBSs conserved between human and mouse. These data were used to build a binary matrix and hierarchical tree, and again, this tree was compared to the expression groups depicted in Figure 2. As in most GO trees, almost every branch of the ConSite tree was significantly associated with an expression group (Fig. 5A). This supports the idea that co-modulation of genes may indeed result from the presence of shared TFBSs in their regulatory regions. To identify putative TFBSs that could be responsible for the observed correlations with expression and thereby potentially identify putative ETV6 partners in transcriptional modulation, we examined the binary table that gave rise to the ConSite tree and selected TFBSs that were detected in the promoter of at least 2 genes of every association group (see Fig. 5B). Some TFBSs were frequently observed, notably Sox-5, Snail, SQUA, c-FOS, bZIP910 as well as E74A, a known ETS TFBS.

Finally, we determined whether any predicted TFBSs were preferentially associated with promoters of ETV6-modulated genes compared to ETV6-non-modulated genes and genes known to be regulated by other human members of the ETS family, regardless of the associated expression profiles. All conserved TFBSs predictions in the promoters of the 66 studied ETV6-modulated genes, 100 ETV6-non-modulated genes and 67 ETS-regulated genes were retrieved using the ConSite software (see Methods for details). For each gene list, the proportion of genes presenting any particular TFBS was calculated and compared between groups in a pairwise fashion using the Fisher’s exact test. Again, FDR was used for multiple testing correction, but note that fewer results remained statistically significant compared to the clustering association studies. CF2-II as well as TFBSs from the REL family (c-REL, NF-kB and p65) were under-represented in the ETV6-modulated group compared to the ETV6-non-modulated genes and NF-kB and p65 were also under-represented in the ETV6-modulated genes vs. ETS-regulated genes (Table 1). We observed an under-representation of Thing1-E47 and RREB-1 among ETV6-modulated genes when compared to the ETV6-non-regulated genes, but statistical significance was not reached (Table 1). Thing1-E47 trend to be over-represented holds also true for ETS members in general (Table 1). Moreover, when compared to ETS-regulated genes, the proportion of ETV6-modulated genes containing Chop-cEBP and NF-Y TFBSs was higher (Table 1), and surprisingly, E74A, a TFBS binding ETS TFs, showed a tendency to be under-represented in the ETV6-modulated group compared to ETV6-non-modulated group (Table 1). Note however that, as opposed to what one would expect, E74A was not over-represented in validated ETS-regulated genes either (Table 1). Also of interest, the abundance of genes containing the two other ETS TFBSs present in the JASPAR database, NRF-2 and SAP-1, showed no significant differences in either group (Table 1). Conserved TFBSs that showed an association using ConSite were also retrieved using the UCSC Genome Browser Tables tool “TFBS conserved” (Karolchik et al. 2004), which uses matrices of the Transfac Matrix Database from Biobase (Wingender, 2004). None of the positive ConSite associations were detected using this approach (data not shown).

## Discussion

To better understand the impact of ETV6 alterations in leukemogenesis, we need to further investigate ETV6-regulated gene expression and initiate the construction of the ETV6-related gene regulation and functional networks. Towards this goal, we recently used an ETV6-inducible system combined with microarray analyses and identified 87 ETV6-modulated genes (Boily et al. 2007). Here, using these putative ETV6 targets, we performed *in silico* exploratory analyses to assess whether genes with similar expression profiles also share either biological or promoter features. Three different clustering methods were used to group the genes according to their expression profiles. Since each method employs a different distance metric, reflecting different yet complementary concepts when used in combination, they should allow for a more stringent analysis of the data (Draghici, 2003). This strategy was proven to be effective given that significant associations were identified for each of the three expression groupings with at least one of the hierarchical trees considered, leading to the identification of specific patterns of co-modulation.

We first attempted to link biological function to the ETV6-modulation profiles on the expectation that co-regulated genes might encode functionally related proteins (Blais and Dynlacht, 2004). We found that many genes closely related by biological features shared a similar expression profile more often than expected by chance suggesting that ETV6 might indeed regulate genes involved in particular functions. Although a number of the GO terms used in the analyses were informative and may even prove to be useful in validating the functions of the ETV6 transcription factor, it should be noted that this approach was limited by the existing gene annotations available in the GO database. Interestingly, the term “cell adhesion” was singled out in the association analysis, a term that is actually quite relevant for ETV6 is known to participate in the cell adhesion process, however other terms such as “cholesterol biosynthesis”, “steroid biosynthesis” and “isoprenoid biosynthesis” or “immune system” that also came out in the clustering analysis have never been linked to ETV6 before.

The combinatorial hypothesis is at the basis of many investigations into the realm of gene transcription and has prompted the development of a number of novel approaches to better understand the complex nature of transcription regulation. This was well illustrated in a study performed in *S. cerevisiae* in which regulatory networks were constructed by searching for combinations of TFBSs within gene promoters and by evaluating the overall similarity of gene expression profiles for any TFBS combinations (Pilpel et al. 2001). Different approaches have also been developed for the identification of TF modules in the promoter of different genes that could potentially be involved in their transcriptional regulation (Klingenhoff et al. 2002). However the characterization of functional TFBSs is still far from being exhaustive because most studies rely on predictions. It has recently been proposed that phylogenetic footprinting might be a suitable approach for decreasing false positive predictions (Lenhard et al. 2003). Using this approach we identified genes presenting similar TFBSs in their promoter region and sharing similar expression profiles thus supporting the combinatorial hypothesis. Taken together these observations support the notion that genes sharing a similar profile of modulation in a particular context can be involved in related biological functions and that particular combinations of TFs may act collectively on the regulatory regions of these genes to modulate their expression in concert, in agreement with the combinatorial hypothesis. The common TFBSs would have been conserved through evolution, what would assure the co-regulation of the genes in given biological pathways or molecular mechanisms thus allowing association between co-modulation and biological features.

Interestingly TFBSs for AML1 and two Sox family members, Sox-5 and SOX17, were found in the promoters of a subset of co-modulated genes that shared the same expression profiles. AML1 was previously shown to interact with members of the ETS family of TFs (Ito, 1999; Lacorazza and Nimer, 2003), which have also been shown to act collectively with Sox4 and Sox2 to regulate transcription (McCracken et al. 1997; Haremaki et al. 2003). In addition, we found that three conserved TFBSs of the REL class, p65, c-REL and NF-kappaB, were under-represented in the promoters of the ETV6-modulated genes relative to ETV6-non-modulated genes. NF-kappaB and c-REL were also under-represented in ETV6-modulated genes compared to ETS-regulated genes. The implications of these findings are not quite clear but they suggest that ETV6-modulated genes are less affected by the NF-kB regulation. Chop-cEBP and NF-Y were over-represented among ETV6-modulated genes compared to ETS-regulated genes, and the TFBS Broad-complex 1 showed a tendency to be over-represented in ETV6-modulated genes compared to ETV6-non-modulated genes. Chop/Gadd153 participate in endoplasmic reticulum stress and cell growth arrest (Oyadomari and Mori, 2004) and can heterodimerize with C/EBP and repress its binding to common class sites but can also bind as a heterodimer to other sites (Ubeda et al. 1996). NF-Y is a CAAT binding TF that acts on a wide range of promoters (Mantovani, 1998). Broad-complex 1 is a Drosophila TF involved in the tissue-specific response to ecdysone (von Kalm et al. 1994). These data warrant additional studies to further characterize the ETV6-modulated gene promoters and to better define the ETV6-related regulatory and functional network.

## Conclusions

In the present study we found that gene expression profiles were often associated with common biological features as well as conserved TFBSs but were poorly associated to promoter sequence similarity. And the biological features that we have described here may in fact be relevant to further understand the functional role of ETV6. Also, our data showed that some TFBSs were under-represented in the promoter regions of ETV6-modulated genes, notably those of the NF-kB family, and that other TFBSs, such as Chop-cEBP and NFY, were over-represented in ETV6-modulated genes compared to ETS-regulated genes as a whole, suggesting that TFs might act in combination with ETV6 to participate in the co-modulation of gene expression. Finally, the exploratory approaches described here should help build upon previous findings of the implications of TFs in transcription regulation and hopefully help direct future study orientations.

## Methods

### Gene lists

The ETV6-modulated gene list was generated from a global expression analysis performed at different time points (48 h) following induction of ETV6 in HeLa cells (Boily et al. 2007). The use of stringent filters allowed us to find 87 ETV6-modulated genes (Boily et al. 2007). These genes and expression data are the basis of the computer-based analyses performed in this paper. Two other gene lists were used for comparative analyses. First, ETV6-non-modulated genes represented a random selection of 100 genes from our microarray analysis with no association with the ETV6 state of expression (ANOVA p-values >0.5): ABCE1, ACAD8, ACCN2, AGER, ALP, ALX4, AMBP, AP4E1, ASCL3, ATP6M, ATP6S14, B4GALT6, BAT2, BMP10, BMP6, CBFB, CD164, CDK6, CDO1, CDR2, CLDN18, COL14A1, COL19A1, CRYGB, CSN10, DCK, DDX16, DOCK3, EMP3, ESR2, FER, FEZ1, FLJ10315, FLJ10512, FLJ12729, FLJ13055, FLOT1, GDF3, GNB1, GOLGB1, GPM6B, GPR85, GPRC5C, GPS1, HSAJ1454, HSPC156, IL13RA1, INPP4A, ITGB4, KARS, KCNK12, KCNMA1, KIAA0573, KIAA0576, KIAA0905, KIAA1039, KIAA1139, KIAA1199, LOC51135, LSAMP, LSM5, MASA, MC5R, MCL1, MDK, MGC3146, MYO1B, NBL1, NCOA1, NDUFB4, NDUFS5, NNAT, NRG1, OAS3, OR6A1, P11, P2RX2, PCLO, POU1F1, PSMD13, RAD23A, RDH5, RHEB2, RPLP2, RPS4Y, RTVP1, SERP1, SH3BGR, SMT3H1, SNAP23, SNRPB, SNTG2, SRR, SYN2, TCF1, TNFSF10, TNXB, UBE2D3, VASP, YME1L1. Second, ETS-regulated genes were a random selection of human ETS TF target genes listed in (Sementchenko and Watson, 2000): BTK, CCND1, CD79B, CD8A, CDC2, CDH5, CSF2RA, DEFA1, DEFA3, EGR1, ENO3, ERBB2, ERF, F3, F9, FCER1A, FES, FLI1, FLT1, GP1BA, GP5, GP9, HBP17, ICAM1, IGJ, IL1B, IL2RB, IL3, IL3RA, IL5, IL8RA, ITGA4, ITGAL, ITGB2, ITGB4, LCK, LCP1, MAGEA1, MCL1, MMP1, MMP3, MSR1, MYB, NCF1, NEFL, NFKB1, NUDT6, PLAU, PPBP, PRF1, PRKCH, PSEN1, RPL32, SCYA5, SCYA7, SERPINB5, SFRS5, SPI1, SPRR1A, SPRR2A, TBP, TGFBR2, TIMP1, TNC, TNFRSF6, VIM, VWF.

### Clustering by gene expression data

Hierarchical clustering of ETV6-modulated genes based on expression data was done using three different methods, each one providing different conceptual information (Draghici, 2003). The first method used data at 0 h, 4 h, 12 h and 48 h and Pearson correlation distances; the second method used the same data but Euclidean distances; whereas the third one considered data at 0 h and 4 h only, with Euclidean distances. With the first method, genes modulated in the same direction under a similar pattern over time were grouped together whereas in the second method, genes modulated in similar intensities over time, but not necessarily in the same direction, are clustered closer to each other. The third method allowed us to consider early modulation events. Genes were assigned to groups based on their proximity in the trees and a colour code was assigned to each group. Most groups were obviously discriminated because their node was separated enough from other nodes to allow unambiguous grouping but in some cases, closely related sub-branches were considered both separated and together in the analyses and different colour codes were assigned to these larger nodes (depicted directly on the tree). All clusterings were performed with the GeneLinker Gold software version 4.0 (Improved Outcomes Software, Inverary, ON, Canada).

### Clustering by Gene Ontology (GO) terms

GO terms corresponding to various organizing principles (molecular function, biological process and cell component), were retrieved from the GO database (Ashburner et al. 2000). Binary matrices were built with genes as one component and GO terms from 1, 2 or 3 organizing principles as the other component. The value 1 was given when a term was assigned to a gene and 0 when it was not. These matrices were used for hierarchical clustering with Pearson correlation distances so that genes sharing a higher number of GO terms were closer together in the hierarchical tree. All clusterings were performed with the GeneLinker Gold software version 4.0 (Improved Outcomes Software, Inverary, ON, Canada).

### Clustering by evolutionary conserved putative transcription factor binding sites (TFBSs)

In each gene, a portion of the DNA sequence corresponding to 3 kb upstream and 0.5 kb downstream of the transcriptional start site from mouse and human (Genbank database) were submitted to ConSite (asp.ii.uib.no:8090/cgi-bin/CONSITE/consite/) (Sandelin et al. 2004) in order to find putative TFBSs conserved between both species. The Orca method (implemented in ConSite) was used for sequence alignment and the analysis was carried out for all individual TFs using the default settings for the conservation cut-off (variable), window size (50) and TF score threshold (80%). A binary matrix was built with genes as one component and all the retrieved TFBSs as the other component. The value 1 was given when a conserved predicted TFBS was found in the particular gene and 0 when it was not. This matrix was used for hierarchical clustering with Pearson correlation distances so that genes sharing a higher number of conserved putative TFBSs were closer together in the hierarchical tree. All clusterings were performed with the GeneLinker Gold software version 4.0 (Improved Outcomes Software, Inverary, ON, Canada).

### Clustering by promoter sequence similarity

When data was available in GenBank, up to one kb of DNA sequence upstream of exon 1 of the ETV6-modulated genes was submitted to Dialign (Morgenstern et al. 1998) for promoter sequence alignment. Dialign (http://bibiserv.techfak.uni-bielefeld.de/dialign/) has the option to give back coordinates, which were used to build a hierarchical tree by using the Phylip program (http://evolution.genetics.washington.edu/phylip.html). Same analysis was also performed using 1.5 kb of alternative promoter sequences (sequences with highest confidence and clone numbers were used) from DBTSS (http://dbtss.hgc.jp/index.html).

### Statistical analyses of gene co-clustering by expression profiles and GO terms, conserved TFBSs or promoter sequence similarity

Genes were grouped based on their expression data and a colour code was assigned to each group (Figs. 1 and 2). Genes were also clustered in hierarchical trees based on their GO terms, their promoter-based conserved TFBSs or their promoter sequence similarities (Fig. 1). In the latter trees, the colour corresponding to the expression group of each gene was displayed on the trees (e.g. Fig. 3). When a colour (expression group) seemed to be over-represented within one of the tree branches by visual observation, the proportion of genes within that branch belonging to the associated expression group was statistically compared to the proportion of genes of the same expression group outside the branch using the Fisher’s exact test. Correction for multiple testing errors was performed using the false discovery rate (FDR) principle.

### Representation of predicted conserved TFBSs in ETV6-modulated genes

The proportion of genes presenting any TFBS in the 5’ regulatory region (3 kb upstream and 0.5 kb downstream of exon 1) of the ETV6-modulated gene list was compared to that of ETV6-non-modulated and ETS-regulated gene lists. All putative TFBSs of the 5’ region that were conserved between mouse and human were retrieved for all the genes from all three lists using ConSite as mentioned above. The proportion of genes sharing a given TFBS as identified by ConSite (JASPAR database) was assessed for each gene list. When these proportions appeared to vary from one gene list to another, they were compared in a pair wise fashion using the Fisher’s exact test to test for statistical significance. Conserved TFBSs found to be differentially represented in any list using ConSite were also probed independently in each list using the Tables Browser tools from the UCSC Genome Browser (http://genome.ucsc.edu/) combined with the Galaxy tools (http://main.g2.bx.psu.edu/) and their proportions in the lists were also statistically compared. FDR was used for multiple testing corrections of probabilities.

## Supplementary Material

Figure 1S. Co-clustering of genes by expression profile and GO terms as well as the frequency of occurrence of the terms involved in the associations **A.** All associations between expression and GO terms (Supplementary Table 1S) were used to build a hierarchical tree in which genes that co-cluster together are found closer together in the tree. **B**. Terms involved in the associations (Supplementary Table 1S) were counted and plotted by frequency of occurrence.

Table 1S. Summary of significant associations found between expression profile and GO term trees.

## Figures and Tables

**Table 1:** Proportion of genes in ETV6-modulated, ETV6-non-modulated and ETS-regulated groups containing conserved predicted TFBSs in their promoter.

	Number of genes (%)			Fisher’s exact test p-value		
Conserved cis-element (ConSite)	ETV6+	ETV6−	ETS+	ETV6+ vs ETV6−	ETV6+ vs ETS+	ETV6− vs ETS+
E74^1^	29 (44)	58 (58)	34 (51)	0.08*	0.49	0.43
Broad-complex_1	13 (20)	9 (9)	9 (13)	0.061*	0.36	0.45
CF2-II	2 (3)	14 (14)	8 (12)	**0.029&**	0.1	0.82
Chop-cEBP	9 (14)	11 (11)	2 (3)	0.47	0.03*	**0.078$**
c-REL	9 (14)	28 (28)	14 (21)	0.036*	0.36	0.36
Hen-1	4 (6)	12 (12)	2 (3)	0.28	0.44	**0.047&**
HFH-1	5 (8)	16 (16)	3 (4)	0.15	0.49	**0.025+**
NF-kappaB	4 (6)	18 (18)	15 (22)	**0.034+**	**0.012+**	0.55
NF-Y	13 (20)	14 (14)	5 (7)	0.39	**0.045+**	0.22
p65	2 (3)	16 (16)	10 (15)	0.0095*	**0.03&**	1.00
RREB-1	0 (0)	5 (5)	1 (1)	**0.076+**	1.00	0.4
Thing1-E47	19 (29)	44 (44)	16 (24)	0.052*	0.56	0.0087*
NRF-2^1^	5 (8)	13 (13)	9 (13)	0.31	0.40	1.0
SAP-1^1^	7 (11)	11 (11)	12 (18)	1.0	0.32	0.25

1 Member of the ETS family of transcription factors. ETV6+: ETV6-modulated genes, ETV6−: ETV6-non-modulated genes, ETS+: ETS-modulated genes. Bold: p-value <0.1, grey-shaded: p-value ≤0.05. FDR correction for multiple testing:

* : significant when FDR = 15%;

+: significant when FDR = 20%;

& : significant when FDR = 25%;

$ : significant when FDR = 30%.
