# Supplementary figures and images for: Connections Between ETV6-Modulated Genes: Identification of Shared Features
Source: Cancer Inform. 2008 Apr 21;6:183–201. doi: 10.4137/cin.s556 (PMC2623305; doi:10.4137/cin.s556)

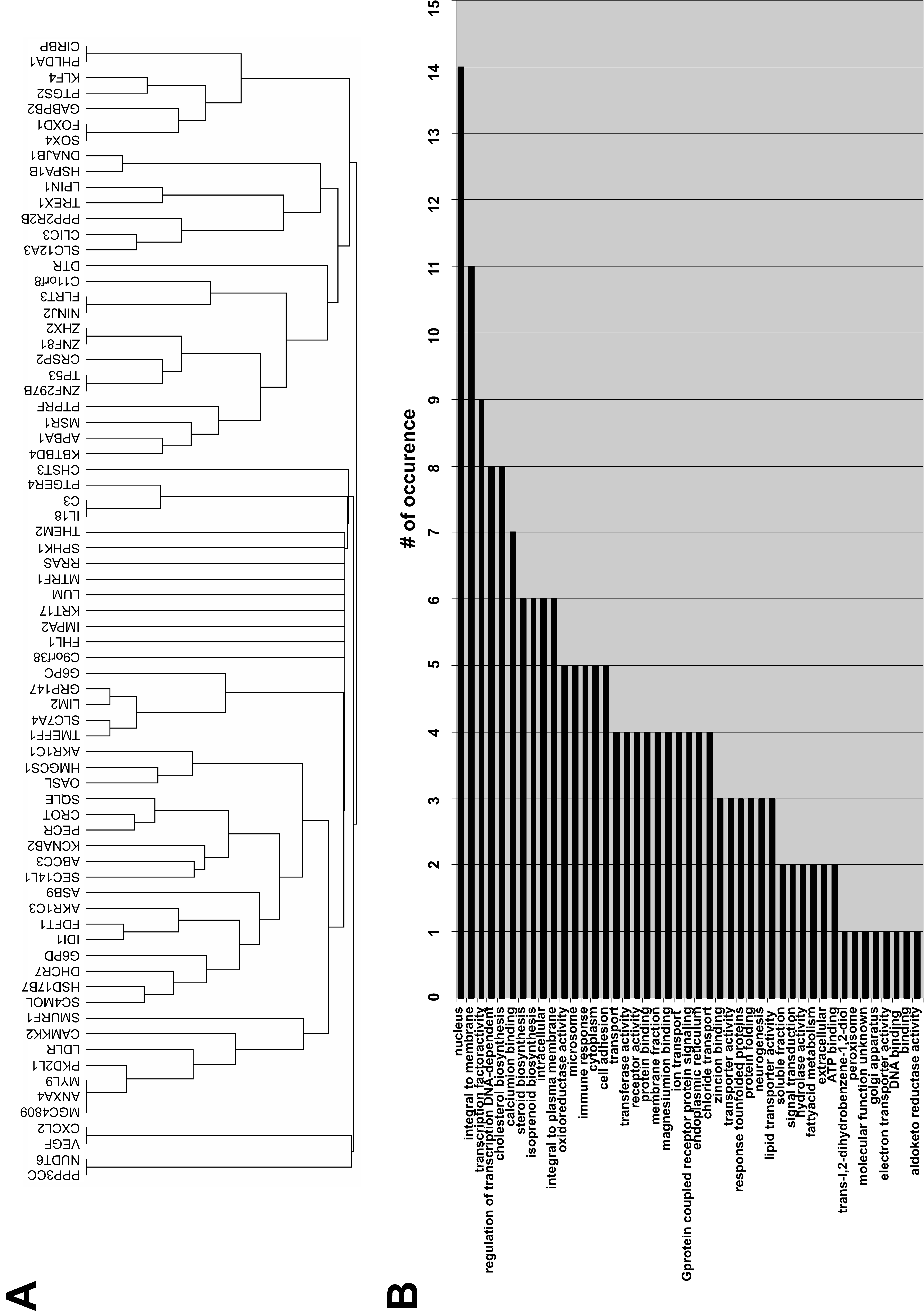

Supplement: Figure 1S. — A. All associations between expression and GO terms (Supplementary Table 1S) were used to build a hierarchical tree in which genes that co-cluster together are found closer together in the tree. B. Terms involved in the associations (Supplementary Table 1S) were counted and plotted by frequency of occurrence. [file cin-6-0183-s1.tif]
