# Supplementary material for: Connections Between ETV6-Modulated Genes: Identification of Shared Features
Source: Cancer Inform. 2008 Apr 21;6:183–201. doi: 10.4137/cin.s556 (PMC2623305; doi:10.4137/cin.s556)
Supplement: Table 1S. [file cin-6-0183-s2.pdf]

Supplementary table

Table 1S - Summary of significant associations found between expression profile and GO term trees.

Hier. Clust. Pearson 0H-48H		Hier. Clust. Euclid. 0H-48H		Hier. Clust. Euclid. 0H-4H	
**All GO terms**					
**Genes**	**Associated terms**	**Genes**	**Associated terms**	**Genes**	**Associated terms**
p= 0.0046*<br>CIRBP<br>FOXD1<br>GABPB2<br>KLF4<br>PHLDA1<br>PTGS2<br>SOX4	-transcription factor activity<br>-regulation of transcription DNA-dependent<br>-nucleus	p= 0.032[+]<br>ABCC3<br>ANXA4<br>CAMKK2<br>G6PC<br>GPR147<br>KCNAB2<br>LIM2<br>MGC4809<br>MYL9<br>PKD2L1<br>SEC14L1<br>SLC7A4<br>TMEFF1	-ATP bonding<br>-intracellular<br>-golgi apparatus<br>-calcium ion binding<br>-integral to membrane<br>-transport<br>-transporter activity	p= 0.016[&]<br>AKR1C1<br>HMGCS1<br>OASL	-transferase activity<br>-cytoplasm
p= 0.028*<br>ABCC3<br>AKR1C1<br>AKR1C3<br>ANXA4<br>CAMKK2<br>CROT<br>DHCR7<br>FDFT1<br>G6PD<br>HMGCS1<br>HSD17B7<br>IDI1<br>KCNAB2<br>MGC4809<br>MYL9<br>OASL<br>PECR<br>PTGER4<br>SC4MOL<br>SEC14L1<br>SQLE	-immune response<br>-ATP binding<br>-intracellular<br>-transferase activity<br>-calcium ion binding<br>-lipid metabolism<br>-cytoplasm<br>-trans-l,2-dihydrobenzene-1,2-diol dehydrogenase<br>-aldo-keto reductase activity<br>-electron transporter activity<br>-binding<br>-fatty acid metabolism<br>-metabolism<br>-sterol biosynthesis<br>-peroxisome<br>-endoplsmic reticulum<br>-integral to membrane<br>-steroid biosynthesis<br>-oxidoreductase activity<br>-isoprenoid biosynthesis<br>-cholesterol biosynthesis<br>-magnesium ion binding<br>-transport<br>-transporter activity<br>-microsome	p= 0.037*<br>APBA1<br>FLRT3<br>KBTBD4<br>LDLR<br>MSR1<br>NINJ2<br>PTPRF	-cell adhesion<br>-protein binding<br>-integral to plasma membrane<br>-receptor activity<br>-lipid transporter activity	p= 0.037[&]<br>CLIC3<br>PPP2R2B<br>SLC12A3	-signal transduction<br>-chloride transport<br>-ion transport<br>-membrane fraction
**GO molecular function terms**					
p= 0.027[+]<br>FOXD1<br>GABPB2<br>KLF4<br>PTPRF<br>SOX4	-protein binding<br>-zinc ion binding<br>-transcription factor activity	p= 0.013*<br>ANXA4<br>LDLR<br>MGC4809<br>MYL9<br>PKD2L1	-calcium ion binding	p= 0.025[+]<br>ANXA4<br>LDLR<br>MGC4809<br>MYL9<br>PKD2L1	-calcium ion binding
**GO biological process terms**					
p= 0.01*<br>DNAJB1<br>HSPA1B	-response to unfolded proteins<br>-protein folding	p= 0.045[+]<br>(0,068)<br>FOXD1<br>SOX4	-regulation of transcription, DNA-dependent	p=0.045[+]<br>(0,079)<br>FOXD1<br>SOX4	-regulation of transcription, DNA-dependent
p=0.032[+]<br>FOXD1<br>GABPB2<br>SOX4	-regulation of transcription, DNA-dependent	p= 0.0045*<br>AKR1C3<br>FDFT1<br>IDI1	-isoprenoid biosynthesis<br>-cholesterol biosynthesis	p= 0.003*<br>DHCR7<br>HMGCS1	-cholesterol biosynthesis
p=0.031[+]<br>ABCC3<br>CROT<br>HSD17B7<br>PECR<br>SC4MOL<br>SEC14L1<br>SQLE	-transport<br>-sterol biosynthesis<br>-fatty acid metabolism<br>-metabolism	p= 0.017*<br>C11orf8<br>FLRT3<br>NINJ2	-neurogenesis<br>-cell adhesion	p= 0.013*<br>C3<br>IL18<br>OASL<br>PTGER4	-immune response<br>-G-protein coupled receptor protein signaling
p= 0.005*<br>AKR1C3<br>DHCR7<br>FDFT1<br>HMGCS1	-lipid metabolism<br>-isoprenoid biosynthesis<br>-cholesterol biosynthesis	p= 0.0093*<br>C3<br>IL18<br>OASL<br>PTGER4	-immune response<br>-G-protein coupled receptor protein signaling		

IDI1<br>LDLR		
**GO cell component terms**		
p= 0.024*<br>CIRBP<br>FOXD1<br>GABPB2<br>KLF4<br>PHLDA1<br>PTGS2<br>PTPRF<br>SOX4<br>-nucleus	p= 0.035[+]<br>ABCC3<br>DTR<br>FLRT3<br>LDLR<br>NINJ2<br>SLC12A3<br>-integral to plasma membrane<br>-membrane fraction	p= 0.0042*<br>ABCC3<br>DTR<br>FLRT3<br>LDLR<br>NINJ2<br>SLC12A3<br>-nucleus
p= 0.0002*<br>CAMKK2<br>SEC14L1<br>SMURF<br>-intracellular	p= 0.0087*<br>DHCR7<br>SC4MOL<br>-endoplasmic reticulum<br>-integral to membrane<br><br>p= 0.014[+]<br>AKR1C1<br>HMGCS1<br>OASL<br>-cytoplasm<br><br>p= 0.041[+]<br>VEGF<br>CXCL2<br>-soluble fraction	p= 0.024*<br>G6PC<br>GPR147<br>SLC7A4<br>TMEFF1<br>-integral to membrane<br><br>p= 0.017*<br>HMGCS1<br>OASL<br>-cytoplasm<br><br>P= 0.014[+]<br>C3<br>IL18<br>-extracellular<br><br>p= 0.041[+]<br>VEGF<br>CXCL2<br>-soluble fraction<br><br>p= 0.03*<br>SMURF1<br>CAMKK2<br>-intracellular
**GO molecular function + biological process terms**		
p= 0.0085*<br>DNAJB1<br>HSPA1B<br>-response to unfolded protein<br>-protein folding<br><br>p= 0.006*<br>ASB9<br>G6PD<br>HSD17B7<br>IDI1<br>SC4MOL<br>-isoprenoid biosynthesis<br>-cholesterol biosynthesis<br>-magnesium ion binding<br>-steroid biosynthesis<br>-oxidoreductase activity<br>-metabolism<br>-sterol biosynthesis<br><br>p= 0.034[+]<br>FOXD1<br>GABPB2<br>KLF4<br>PTPRF<br>SOX4<br>-transcription factor activity<br>-regulation of transcription, DNA-dependent<br>-protein binding	p= 0.031*<br>CROT<br>HMGCS1<br>OASL<br>-transferase activity<br><br>p= 0.028*<br>ABCC3<br>G6PC<br>SEC14L1<br>SLC7A4<br>-transport<br>-transporter activity<br><br>p= 0.0063*<br>CLIC3<br>PPP2R2B<br>SLC12A3<br>-signal transduction<br>-chloride transport<br>-ion transport<br><br>p= 0.0063*<br>FDFT1<br>IDI1<br>-isoprenoid biosynthesis<br>-cholesterol biosynthesis<br>-magnesium ion binding<br><br>p= 0.0056[+]<br>DHCR7<br>G6PD<br>SC4MOL<br>-oxidoreductase activity<br><br>p= 0.0084*<br>ANXA4<br>LDLR<br>LPIN1<br>MGC4809<br>MYL9<br>PKD2L1<br>SMURF1<br>-molecular function unknown<br>-calcium ion binding<br><br>p= 0,0084*<br>ABPA1<br>CRSP2<br>FLRT3<br>KBTBD4<br>MSR1<br>NINJ2<br>ZHX2<br>ZNF297B<br>ZNF81<br>-zinc ion binding<br>-transcription factor activity<br>-regulation of transcription, DNA-dependent<br>-cell adhesion<br>-protein binding<br>-neurogenesis<br>-DNA binding<br>-receptor activity<br><br>p= 0,031*<br>C3<br>IL18<br>PTGER4<br>-immune response<br>-G-protein coupled receptor protein signaling	p= 0.006*<br>FDFT1<br>G6PD<br>IDI1<br>SC4MOL<br>-isoprenoid biosynthesis<br>-cholesterol biosynthesis<br>-magnesium ion binding<br>-oxidoreductase activity<br><br>p= 0.006*<br>FOXD1<br>SOX4<br>-transcription factor activity<br>-regulation of transcription, DNA-dependent<br><br>p= 0.013*<br>C3<br>IL18<br>PTGER4<br>-immune response<br>-G-protein coupled receptor protein signaling
**GO molecular function + cell component terms**		

p= 0.009*<br>CIRBP<br>FOXD1<br>KLF4<br>PHLDA1<br>PTGS2<br>SOX4<br>-nucleus<br>-transcription factor activity	p=0.0035*<br>CIRBP<br>PHLDA1<br>-nucleus	p= 0.0035*<br>CIRBP<br>PHLDA1<br>-nucleus
p= 0.011*<br>NUDT6<br>PPP3CC<br>-hydrolase activity<br><br>p= 0.0003*<br>GPR147<br>TMEFF1<br>LIM2<br>SLC7A4<br>-integral to membrane<br><br>p= 0.027*<br>DHCR7<br>FDFT1<br>HSD17B7<br>SC4MOL<br>-endoplasmic reticulum<br>-integral to membrane<br>-oxidoreductase activity<br><br>p= 0.011*<br>SEC14L1<br>SMURF1<br>-intracellular	p= 0.0059*<br>FOXD1<br>SOX4<br>-nucleus<br>-transcription factor activity<br><br>p= 0.022*<br>DTR<br>MSR1<br>-integral to plasma membrane<br>-receptor activity<br><br>p= 0.0015*<br>GPR147<br>LIM2<br>SLC7A4<br>TMEFF1<br>-integral to membrane<br><br>p= 0.0047*<br>ANXA4<br>CAMKK2<br>LDLR<br>MGC4809<br>MYL9<br>PKD2L1<br>-calcium ion binding	p= 0.011*<br>ZNF81<br>ZHX2<br>-nucleus<br>-zinc ion binding<br>-transcription factor activity<br><br>p=0.0059*<br>FOXD1<br>SOX4<br>-nucleus<br>-transcription factor activity<br><br>p= 0.022*<br>NUDT6<br>PPP3CC<br>-hydrolase activity<br><br>p= 0.011*<br>DTR<br>MSR1<br>-integral to plasma membrane<br>-receptor activity<br><br>p= 0.0028*<br>HMGCS1<br>OASL<br>-transferase activity<br>-cytoplasm<br><br>p= 0.011*<br>C3<br>IL18<br>-extracellular<br><br>p= 0.0084*<br>GPR147<br>LIM2<br>SLC7A4<br>TMEFF1<br>-integral to membrane<br><br>p= 0.025*<br>ANXA4<br>CAMKK2<br>LDLR<br>MGC4809<br>MYL9<br>PKD2L1<br>-calcium ion binding
**GO biological process + cell component terms**		
p= 0.0012*<br>CIRBP<br>FOXD1<br>GABPB2<br>PHLDA1<br>SOX4<br>-nucleus<br>-regulation of transcription, DNA-dependent<br><br>p= 0.012*<br>DNAJB1<br>HSPA1B<br>-response to unfolded protein<br>-protein folding<br>-nucleus<br><br>p= 0.024*<br>DHCR7<br>FDFT1<br>HSD17B7<br>SC4MOL<br>-metabolism<br>-endoplasmic reticulum<br>-integral to membrane<br>-steroid biosynthesis<br>-cholesterol biosynthesis<br><br>p= 0.0038*<br>CHST3<br>PKD2L1<br>-integral to membrane<br><br>p= 0.018*<br>GPR147<br>LIM2<br>TMEFF1<br>-integral to membrane<br><br>p= 0.0004*<br>CAMKK2<br>SEC14L1<br>SMURF1<br>-intracellular	p= 0.0013*<br>CIRBP<br>PHLDA1<br>-nucleus<br><br>p= 0.0064*<br>CLIC3<br>SLC12A3<br>-chloride transport<br>-ion transport<br>-membrane fraction<br><br>p= 0.014*<br>C11orf8<br>FLRT3<br>NINJ2<br>-neurogenesis<br>-cell adhesion<br>-integral to plasma membrane	p= 0.0013*<br>CIRBP<br>PHLDA1<br>-nucleus<br><br>p= 0.0051*<br>CLIC3<br>CRSP2<br>FLRT3<br>HSPA1B<br>LPIN1<br>NINJ2<br>SLC12A3<br>TREX1<br>-chloride transport<br>-ion transport<br>-membrane fraction<br>-nucleus<br>-cell adhesion<br>-integral to plasma membrane

FDR correction for multiple testing: *: significant when FDR= 5%; +: significant when FDR= 10%; &: significant when FDR= 15%.
